# Empowering districts to target priorities for improving child health service in Uganda using change management and rapid assessment methods

**DOI:** 10.3402/gha.v9.30983

**Published:** 2016-05-24

**Authors:** John Odaga, Dorcus K. Henriksson, Charles Nkolo, Hector Tibeihaho, Richard Musabe, Margaret Katusiime, Zaccheus Sinabulya, Stephen Mucunguzi, Anthony K. Mbonye, Joseph J. Valadez

**Affiliations:** 1 Liverpool School of Tropical Medicine, Kampala, Uganda; 2 Health Systems and Policy (HSP) Research Group, Department of Public Health Sciences, Karolinska Institutet, Stockholm, Sweden; 3 International Maternal and Child Health Department, Uppsala University, Uppsala, Sweden; 4 ChildFund International, Kampala, Uganda; 5 Ministry of Health, Kampala, Uganda; 6 School of Public Health, College of Health Sciences, Makerere University, Kampala, Uganda

**Keywords:** LQAS, Bottleneck analysis, priority setting, CODES

## Abstract

**Background:**

Local health system managers in low- and middle-income countries have the responsibility to set health priorities and allocate resources accordingly. Although tools exist to aid this process, they are not widely applied for various reasons including non-availability, poor knowledge of the tools, and poor adaptability into the local context. In Uganda, delivery of basic services is devolved to the District Local Governments through the District Health Teams (DHTs). The Community and District Empowerment for Scale-up (CODES) project aims to provide a set of management tools that aid contextualised priority setting, fund allocation, and problem-solving in a systematic way to improve effective coverage and quality of child survival interventions.

**Design:**

Although the various tools have previously been used at the national level, the project aims to combine them in an integral way for implementation at the district level. These tools include Lot Quality Assurance Sampling (LQAS) surveys to generate local evidence, Bottleneck analysis and Causal analysis as analytical tools, Continuous Quality Improvement, and Community Dialogues based on Citizen Report Cards and U reports. The tools enable identification of gaps, prioritisation of possible solutions, and allocation of resources accordingly. This paper presents some of the tools used by the project in five districts in Uganda during the proof-of-concept phase of the project.

**Results:**

All five districts were trained and participated in LQAS surveys and readily adopted the tools for priority setting and resource allocation. All districts developed health operational work plans, which were based on the evidence and each of the districts implemented more than three of the priority activities which were included in their work plans.

**Conclusions:**

In the five districts, the CODES project demonstrated that DHTs can adopt and integrate these tools in the planning process by systematically identifying gaps and setting priority interventions for child survival.

## Introduction

In Uganda, local health system managers have the responsibility for health service delivery including setting priorities and managing resources (1). Managers in these settings are often constrained by availability of field-friendly tools to guide them on how to maximise benefits using limited resources (2). However, some tools that are based on economic principles of opportunity cost and marginal benefit (3) exist to help this process. These include the burden of disease and cost-effectiveness analysis (4), The Marginal Budgeting for Bottlenecks tool (5), WHO-CHOICE (Choosing Interventions that are Cost-Effective) (6, 7), Lives Saved Tool (8), and small-sample data collection through Lot Quality Assurance Sampling (LQAS) (9).

Widespread use of tools such as those mentioned earlier is limited by unavailability, poor quality of data, poor knowledge of the tools, and poor adaptation to local contexts (3). Consequently, health managers in low-income countries commonly base funding decisions on what has been funded previously, or what is prioritised by the ministry(s) of health or donors (3, 10). These priorities are often based on national or international indicator values, rather than on locally available evidence (11). They also tend to provide little guidance for maximising health benefit with limited resources (3, 10).

In Uganda, the responsibility for delivery of basic health services is devolved to District Local Governments through District Health Teams (DHTs) (1). Basic health services are provided through a referral structure consisting of Village Health Teams (VHTs comprised of community volunteers) and health facilities (HFs, including Health Centres II, III and IV, and hospitals).

The bulk of funding at the district level is through government grants that are usually earmarked, leaving managers minimal ‘fiscal space’ for reallocation of resources (12). Another source of funding is through non-governmental organisations (NGOs), but because most of this funding is often managed vertically, it is not always directed towards programmes consistent with the district priorities.

Given the above scenario, the Community and District Empowerment for Scale-up (CODES) project aimed to provide a set of tools that were used to aid contextualised priority setting, fund allocation, and problem-solving in a systematic way, so as to improve effective coverage and quality of child survival interventions. This paper presents the compendium of tools and interventions that were used in the proof-of-concept phase in Mukono, Masaka, Wakiso, Bukomansimbi, and Buikwe districts in Uganda.

### The CODES project

The CODES (13, 14) project is a 5-year project that tests a district-focused health systems management strategy that aims at strengthening district priority setting by combining the monitoring of key population-based indicators, quality of care, and community engagement. The intention is that these three components together result in improved, equitable coverage, and quality of key interventions for children under 5 years of age (U5s). This outcome is expected to reduce childhood (U5) illness and death due to diarrhoea, malaria, and pneumonia. The CODES project combines tools designed to systematise identification of gaps, priority setting, allocation of resources, and problem-solving. The project also empowers and engages communities in monitoring health service provision and to demand quality services through community dialogues based on Citizen Report Cards (CRC) and U reports as a feedback mechanism.

The tools include LQAS, Bottleneck analysis (BNA) using the Tanahashi model (15), Causal analysis, and Continuous Quality Improvement, which are the supply-side tools; and community dialogues based on CRC and U reports, which are the demand-side tools. This paper mainly focuses on the supply-side tools, which are used by the service providers. They combine the use of local evidence to identify and prioritise child survival interventions with the lowest coverage and quality, and then use analytic approaches to identify district-specific bottlenecks to scaling-up, what is causing them, what are the possible solutions, and prioritising these solutions with the aim of increasing coverage and quality of care. The tools enable managers to identify the worst-performing subdistrict areas (subcounties) such that they are prioritised. Learning and using of tools is promoted through training, participation, and learning networks (peer-to-peer learning) and through mentoring.

## Methods

### Study setting

This study was conducted in five districts during the proof-of-concept phase. The criteria used by the CODES project to select the districts included high child mortality rates and the representation of both new and old districts (16). The old districts are Masaka, Mukono, and Wakiso and the new districts are Bukomansimbi and Buikwe.

### Interventions

#### Lot Quality Assurance Sampling surveys

##### Indicators

In total, 151 indicators drawn from three levels, that is, the Community (Household), HF, and VHT were used (55 in the community/household surveys, 35 in the HF surveys, and 28 in the VHT surveys; Table 1). They were drawn from both the WHO/UNICEF child survival indicators and Uganda Ministry of Health services indicators and strategies.

**Table 1:** Focus area for survey indicators

Community survey	Health facility survey	Village Health Team (VHT) survey
Antenatal care and delivery	Staffing levels	VHT training
Immunisation of the children	Availability of drugs and supplies used for the treatment of pneumonia, diarrhoea, and malaria in U5's	Handwashing promotion
Infant feeding	New-born and child care	Key family care practices
Vitamin A supplementation	Facility-based care using recommended treatment for pneumonia, diarrhoea, and malaria	VHT referral
HIV prevention including Prevention of Mother To Child Transmission of HIV/AIDS (PMTCT)	Access to treatment for pneumonia, diarrhoea, and malaria	Availability of drugs used for the treatment of diarrhoea, pneumonia, and malaria
Integrated Community Case Management of childhood illnesses (ICCM)	Health facility infrastructure	VHT knowledge of danger signs
Water supply	Health information system and reporting	VHT coverage and intervention activities
Hand washing practices	Payment for health services	Oversight and equipment given to VHTs
Latrine coverage	Training of the health service providers	
Healthcare seeking behaviour and treatment for pneumonia, diarrhoea, and malaria	Health worker supervision	
Caregiver knowledge of child danger signs	Health service guidelines (protocols)	
Prevalence of pneumonia, diarrhoea, and malaria	Referral of clients from the community to health facility	
Long-Lasting Insecticide treated mosquito Net (LLIN) coverage, ownership and usage	Health worker performance assessment for treatment of sick child and counselling the mother/caretaker	

##### Survey questionnaire

The HF and Community LQAS survey questionnaires were developed based on the agreed survey indicators. They were pretested and adjustments were made where necessary. The HF questionnaire contained an introductory/consent page and four modules. Module 1 covered clinical observations of six children, module 2 covered six exit interviews, module 3 covered HF checklist, and module 4 covered health worker interviews and record reviews. The LQAS community questionnaire largely followed the indicator categories as shown in Table 1.

##### Training of data collectors

Data collectors for the HF questionnaire were staff from the respective districts. Each data collection team consisted of a clinical officer and a nurse, and these were supervised by a district supervisor. Data collectors were trained for 2 days following a practical approach on the survey questionnaire. The data collectors for the community LQAS survey were also drawn from the district health, education, and community departments. Each district had a team of 10 trained data collectors, that is, a pair of data collectors per district and a district supervisor.

##### Sampling frame and sample size

The sampling frame for the HFs consisted of all HC IIIs, HC IVs, and district hospitals within the five districts. Both government-owned and private not-for-profit HFs were enrolled in the study. Health centre IIs were excluded because of their limited services delivery. The LQAS hypergeometric calculator was used to determine the number of HFs to survey in each district. The calculations are based on the following assumptions:

The desired performance threshold (pU); at least 80% of HFs in each district were expected to demonstrate adequate performance for each specific indicator included in the assessment.

- A lower threshold below which performance will be deemed highly unacceptable, set at 50%.
- The probability of misclassifying a district with high performance as having low performance (α error) was set at <0.10.
- The probability of misclassifying a district with low performance as high (β error) was set at <0.10.

Facilities visited were selected from the district sampling frame using Simple Random Sampling With-out Replacement (SRSWoR) (17). The HF clinical observations were selected randomly using SRSWoR and these were the same cases that were used for the exit interview.

For the community LQAS, the district was subdivided into Supervision Areas (SAs) based on subcounties. In areas where SAs were carved out of multiple subcounties, these were fairly homogeneous based on the study indicators and shared boundaries. It was also ensured that the SA had similar and comparable populations. Probability Proportional to Size Sampling was used to select the villages where interviews were conducted. A sample of 19 locations for interviews was selected for each SA. Data from the survey were also used to classify SAs into either high or low performance so that the available limited resources could be targeted where they were needed the most.

##### Data management

Data were collected by trained data collectors who worked in pairs and were supervised by a district supervisor. Each complete questionnaire was reviewed for correctness and completeness by the data collector and two supervisors; one at the district and the overall project supervisor.

A computerised database following the structure of the questionnaire was developed in EPIDATA. The database was tested for accuracy and consistency before entry commenced. A double data entry system was used. The first set of entries was done at the district, and this was meant to build capacity of the district personnel in data management for sustainability. The second data entry round was done by the project staff. Data from these two sets were compared and entries for one set corrected/cleaned before it was exported into SPSS and analysed.

#### Bottleneck analysis

**Fig. 1:**
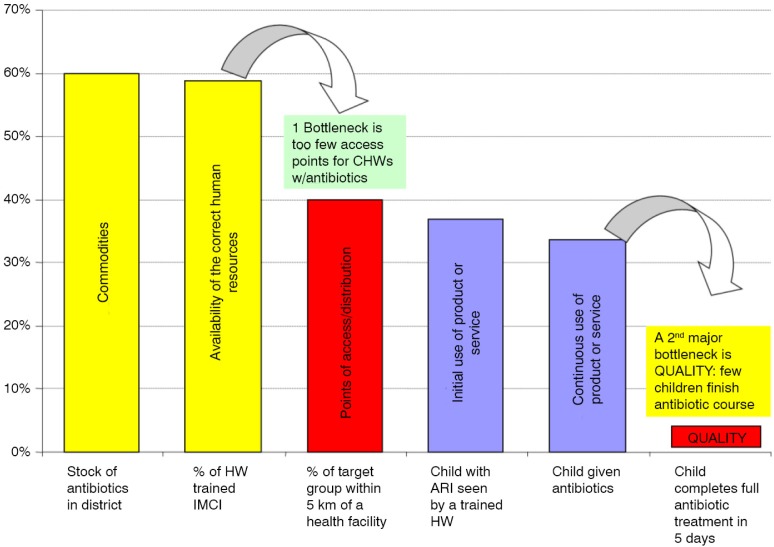
The Tanahashi model of health systems’ bottlenecks. Source: Adapted by O'Connell from Tanahashi, 1978.

Data from HF and LQAS surveys were aggregated at district level and the results were expressed in the form of percentage coverage and used to populate the selected indicators. The indicators showing coverage were fed into a BNA tool, which generates coverage outputs according to the Tanahashi model (15) as shown in Fig. 1. The Tanahashi model is a graphical display of six health systems’ factors, which interact to influence the effective coverage of key child survival indicators. The model organises these factors in a logical manner into supply-side and demand-side factors in order to assess health system constraints. The three supply-side factors are availability of essential commodities, availability of human resources who are appropriately trained to provide the interventions under review, and the proportion of the target population who have access to the intervention (who are within a 5-km radius of the facility or a health worker offering the intervention). The demand-side factors include extent of initial utilisation of an intervention by the target population, continued utilisation, and the level of quality coverage (the proportion of the target population who receive the intervention as per relevant guidelines).

Outputs from the Tanahashi model were then presented to the DHTs, planners, and policymakers. The district teams were facilitated to identify and prioritise at least five key gaps (Table 2) affecting quality delivery of interventions for managing diarrhoea, pneumonia, malaria, and immunisation at the HF and community levels, which is the VHT in their districts.

**Table 2:** An example of gaps in the health system; possible causes and solutions identified by a district health team

Bottlenecks			
		
Type of bottleneck	Description	Causes of common bottlenecks	Proposed solutions/activities
Human resource	Staff not trained in the new strategies to manage diarrhoea	District does not have capacity to train staff in Integrated Management of Childhood Illnesses (IMCI)	• Conduct training of trainers in IMCI for the district • Then train other health workers in the district
	Staff not refreshed in the IMCI strategy	Refresher trainings in IMCI and Integrated Community Case Management (iCCM) have not been held in the district for a long time	Give refresher trainings to those trained earlier
	VHTs not trained to manage diarrhoea at community level	No VHTs have ever been given basic or iCCM training	• Conduct training of trainers in iCCM for the district • Train VHTs in iCCM
Commodities (ORS and zinc)	Essential commodities (ORS and zinc) not listed on pre-order requisition forms	Zinc is not listed among the essential commodities, which are routinely supplied to the district by National Medical Stores (NMS) and Joint Medical Stores (JMS)	• Acquire/order zinc for health facilities • Advocate for inclusion of zinc on the Push drugs list by the National Medical Stores
Effective coverage	Zinc not on the list of medicines provided to sick children	District does not have capacity to procure essential commodities (ORS and zinc)	Community sensitisation and health education for mothers to seek treatment early and to demand for ORS and zinc

#### Causal analysis

After completion of the BNA process, the CODES project staff facilitated the Causal analysis process in each district during which time DHTs from the respective districts critically explored the likely causes of the major bottlenecks identified and proposed solutions for overcoming them. During this process, the districts used their working knowledge and other sources of data, for example, Health Management Information System, Demographic Health Survey, and district or national surveys like the Malaria Indicator Survey among others. Causal analysis was aided by the UNICEF management checklist, which consists of an algorithm for identifying key managerial shortcomings that might be responsible for the observed bottlenecks. The Causal analysis process was also supported by data derived from community dialogues with community members and HF staff. Community dialogues were facilitated by another implementing partner Advocates Coalition for Development and Environment, which focuses on demand-side interventions (13). For each identified cause (factor responsible for the observed gap in coverage), the district teams identified possible solutions. Emphasis was placed on identifying managerial short comings – or interventions which the DHT could implement themselves or have direct influence on. Potential solutions were further prioritised using a rank-scoring approach that takes into account effectiveness of the suggested intervention, feasibility, affordability, and acceptability. The tool used in ranking potential solutions is shown in Table 3.

**Table 3:** Tool used in rank-scoring potential solutions to health systems bottlenecks

	Evidence	Feasibility (policy, capacity)		Affordability (cost-effectiveness and availability of funding)		Acceptability				
							
Identified solutions	Is there evidence of its effectiveness?	Approved supportive policy	Do the HS have capacity to implement?	Is the solution cost-effective?	Is funding available?	Is it acceptable to the stakeholders?	Is it acceptable communities?	Equity focused: Does it benefit the poor?	Score	Recommendations
Solution 1	1 2 3	1 2 3	1 2 3	12 3	1 2 3	1 2 3	1 2 3	1 2 3		
Solution 2	1 2 3	1 2 3	1 2 3	12 3	1 2 3	1 2 3	1 2 3	1 2 3		
Solution 3	1 2 3	1 2 3	1 2 3	12 3	1 2 3	1 2 3	1 2 3	1 2 3		

#### Workplan

Prioritised activities were then incorporated into district annual health operational plans, which were then costed and financed through central government grants and district local revenues. Sometimes, this entailed reallocating funds from other activities to the identified priorities. Some of the activities were financed by NGOs in the districts and unfunded priorities were usually carried forward into the next financial year whereas some of the identified interventions were included in the district development (strategic) plans.

#### Monitoring and mentoring

Implementation of activities was monitored through routine follow-up visits by the project staff during which good practices were documented, constraints identified, solutions generated, and technical guidance provided through mentorship.

#### Peer-to-peer learning

Annual peer-to-peer workshops brought together DHTs from the project districts to share experiences on the progress of implementation of identified activities. During these workshops, success factors were identified and lessons learnt shared especially on how to address some cross-cutting constraints.

### Ethical considerations

Ethical clearance was obtained from Uganda National Council for Science and Technology (UNCST-SS 2548) to conduct this study. Permission to conduct the study was also sought from the District Health office in all the five districts. Individual consent was obtained from all the participants prior to being interviewed.

## Results

This section uses data from one district to demonstrate how a combination of local evidence and a set of interventions and tools described in the methods section can be used to guide the process of priority setting and resource allocation for the management of childhood diseases. Additionally, this paper presents evidence from three districts^1^ to show the budget impact of this process.

### Identifying the weakest interventions and classifying performance of SAs

The performance of five SAs in one of the districts based on selected indicators in which the desired performance threshold was set at 80%, and the lower unacceptable level at 50% is shown in Table 4. The table shows the number of children out of a sample of 19 in each SA that were assessed and had a desired outcome, for example, the number of children who were treated with Oral Rehydration Salts (ORS) and zinc. LQAS uses a sample of 19 because it is found to be the smallest most precise sample size. However, computing a proportion based on it would yield a less precise estimate because of its confidence interval. However, a proportion (district average) computed at district level after collating results from the five SAs would offer a usable estimate.

**Table 4:** District-wide coverage of interventions and performance of SAs as judged by LQAS-based decision rules

Indicators	District average (%)	SA 1	SA 2	SA 3	SA 4	SA 5	DR
Number of children under 5 years with cough and fast/difficult breathing in the last 2 weeks who were treated with antibiotic according to national policy within 24 h of onset of symptoms	58.6%	10 Low	8 Low	13 High	10 Low	14 High	13
Number of children under 5 years with diarrhoea in the last 2 weeks who were treated with ORS and zinc supplements within 24 h of the onset of symptoms	4.0%	1 Low	2 Low	0 Low	0 Low	1 Low	13
Number of children under 5 years with confirmation of malaria diagnosis who received treatment with a national recommended ACT within 24 h of the onset of symptoms	7.8%	1 Low	2 Low	0 Low	4 Low	1 Low	13

Table 4 shows that by applying LQAS decision rules, two of the five SAs (SA 3 and SA 5) with scores of 13 and 14, respectively, can be judged as being high performers on account of how well health workers managed children with symptoms of acute respiratory illnesses in relation to national treatment guidelines. With regard to the same indicator, performance in the rest of the SAs (SA 1, SA 2, and SA4) is judged as unacceptably low and should be prioritised for intervention in the face of limited resources. Applying the same performance standards, LQAS principle classifies performance of all SAs as unacceptably low with regard to the last two indicators. That is, implementation of each of the last two interventions was weak for all the SAs. Hence, it is difficult to prioritise resources in this situation. This principle of LQAS-based classification of SAs was carried out in each district for all the 151 indicators in order to identify the weakest interventions and the worst-performing SAs.

### Identifying the main bottlenecks

**Fig. 2:**
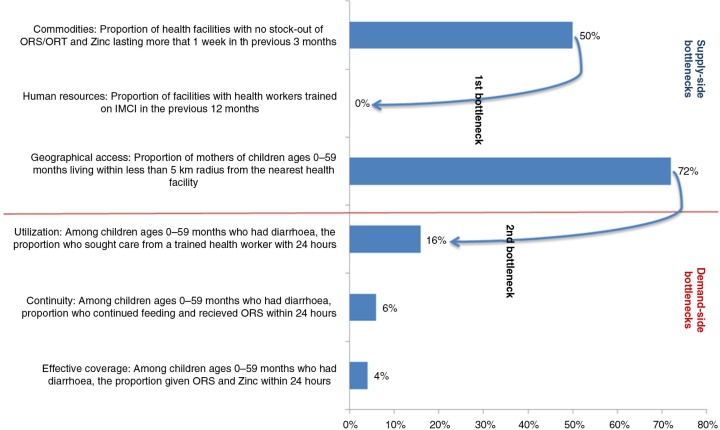
A Tanahashi model showing health systems’ factors related to treating a child with diarrhoea.

The following section uses the data on management of diarrhoea to illustrate the process of Bottleneck and Causal analysis. Fig. 2 is a graphical representation using the Tanahashi model of some of the bottlenecks in the health system that may influence the coverage of quality management of diarrhoea in the district.

The figure shows that of all the children 0–59 months of age who were reported to have had diarrhoea, only 4% received appropriate treatment (effective coverage). The figure also shows that, despite the availability of ORS in most HFs in the district, there was shortage of appropriately trained staff to manage children with diarrhoea (supply-side constraint). Secondly, few children with diarrhoea sought care from a trained health worker (poor health-seeking behaviour – demand-side constraint), despite the high level of geographical accessibility to HFs. Therefore, low effective coverage of treatment using ORS and zinc could have resulted from interplay between these two factors (few appropriately trained health workers and poor health-seeking behaviour).

By means of these graphs, the DHTs were able to identify key bottlenecks for each intervention studied. This then paved the way to identify the corresponding root causes for the barriers and possible solutions.

### Causal analysis

Table 2 is a sample of bottlenecks identified, the root causes of the bottlenecks, and the suggested solutions to address the cause with regard to managing a child with diarrhoea. These factors (causes) represent managerial constraints that DHMTs themselves can address at their level. In this example, the district team identified shortage of essential commodities and inadequate number of trained staff as the major bottlenecks to effective management of a child with diarrhoea. Poor health-seeking behaviour was not specifically identified by this team as one of the major health systems constraints. However, the team proposed an intervention to improve health-seeking behaviour. Such scenarios were identified during mentorship and were addressed so that proposed solutions are consistent with the identified bottlenecks and their causes.

### Work plans and resource allocation

The activities identified during the Bottleneck and Causal analyses were later included in the district's health operational work plan.

For the fiscal year 2012–2013, the DHTs in the five districts identified and prioritised child survival oriented interventions worth an average of US$83,142, representing 18% of the total recurrent non-wage budget; 52% of this was funded through government allocation. NGOs committed to fund 31% of the budget for child survival priority interventions, through reallocation from other activities. NGO funding or commitments were achieved following stakeholder meetings at the district level, where funding gaps were discussed. UNICEF provided an additional US$10,000 to each of the five districts to finance some of the short falls, which amounted to 12% of the child survival budget. Five percent of the budget remained unfunded, consisting of activities for which funding had been not been committed.

### Adoption

Early implementation experiences from the project (13) indicate that district teams readily adopted the interventions described above. All five districts have operationalised the intervention package and perceived it as useful (13). All DHTs were able to develop two district health operational work plans, which are based on the evidence from the BNA and Causal analysis tools (FY 2012–2013 and FY 2013–2014 work plans). Each of the five districts has implemented more than three of the priority activities, which were included in their work plans.

## Discussion

For the first time in Uganda, health planners at the district level have been provided with tools that can enable them to systematically prioritise interventions during the planning process and to target limited resources where they are most needed. With the use of these tools, analyses are informed by local evidence, thereby allowing decisions to be context specific. Use of LQAS-based data helps in identifying the worst-performing areas such that resources are targeted to these areas. LQAS is sensitive to detecting poorly performing parts of the system being sampled, thus aiding local managers to identify components of the system that require urgent action and to prioritise resources (9). LQAS-based methods have recently been applied extensively in developing countries to assess child survival and maternal and child health interventions (9, 18, 19), in monitoring malaria epidemics and in assessing communities for Schistosoma (20). Its main advantages are that it requires a small sample size, is rapid, and therefore it is not resource-intensive (18, 21, 22). The framework also helps identify interventions that are generally performing poorly across the district.

The BNA and Casual analysis enabled district managers to focus more specifically on particular interventions and therefore identify the gaps in service provision. These specific focuses allowed for a more in-depth analysis of the health system for particular interventions and with the casual analyses, solutions were identified. As the bottlenecks affecting a particular intervention can be generalised to represent those affecting similar interventions within the health system (11), analysis for one intervention could lead to wider improvements in the health system as a whole.

An important advantage of this approach is the participatory nature, whereby district-based decision makers plan together and hence district team members have an opportunity to reflect together (13) on their major constraints and on the best possible solutions to address them.

Additionally, the CODES project consists of a number of learning platforms (peer-to-peer learning, collaborative learning sessions) (13, 14). These platforms have proved to be beneficial in offering mutual support and in rapidly transferring new ideas and management approaches between districts.

Furthermore, by actively engaging members of the DHT in every aspect and step of the project, CODES has built capacity at the district level in designing and implementing large-scale research, and in utilising the data thereof, to achieve allocative efficiency. There are increased and informed debates among the DHTs on how to identify and address district priorities (13).

The CODES project has enabled districts to clearly define their unfunded priorities, based on a systematic approach. This information has been successfully used by a number of district leaders from the project districts to raise funds from other development partners and donors operating in those districts (13).

### CODES project has some shortcomings

Causal analysis is based on perceived causes of bottlenecks, rather than on empirical evidence. Therefore, the proposed solutions may be characterised by some margin of error. However, the Causal analysis is based on team work and consensus; therefore, the amount of error associated with identified causes of bottlenecks and the suggested solutions may be marginal.

Local evidence is based mainly on community and HF survey data. Although LQAS-based surveys are relatively cheaper than cluster surveys, the cost associated with these (LQAS) surveys are still high. The project is exploring ways of collecting community and facility-based data by piggy-backing data collection process on routine supervision visits.

## Conclusion

The ‘proof-of-concept’ phase of the CODES project has shown that district health planners in Uganda can adopt and integrate the UNICEF set of tools for priority settings and be able to make decisions at the margin, as long as such methods are contextualised, user-friendly, and participatory. Therefore, these methods could be scaled-up to other districts in Uganda, and similar developing countries. Active participation of district teams, use of local evidence, and learning networks appear critical in a successful diffusion of CODES intervention.

## Footnotes

1 With complete and well disaggregated data.

## Paper context

Local health system managers in low income countries like Uganda are often constrained by availability of field friendly tools to guide them on maximizing benefits using limited resources. This study shows that district health planners in Uganda can adopt and integrate of tools for priority settings to make decisions at the margin, as long as such methods are contextualized, user-friendly, and participatory. Therefore, these methods could be scaled-up in Uganda, and similar developing countries.
